# Platelet receptor polymorphisms do not influence *Staphylococcus aureus*–platelet interactions or infective endocarditis

**DOI:** 10.1016/j.micinf.2010.10.016

**Published:** 2011-03

**Authors:** Shruti Daga, James G. Shepherd, J. Garreth S. Callaghan, Rachel K.Y. Hung, Dana K. Dawson, Gareth J. Padfield, Shi Y. Hey, Robyn A. Cartwright, David E. Newby, J. Ross Fitzgerald

**Affiliations:** a Center for Infectious Diseases and The Roslin Institute, Royal (Dick) School of Veterinary Studies, University of Edinburgh, Chancellor’s Building, Edinburgh EH16 4SB, United Kingdom; b Center for Cardiovascular Science, University of Edinburgh, Chancellor’s Building, Edinburgh EH16 4SB, United Kingdom

**Keywords:** Genetic polymorphisms, Platelet aggregation, Endocarditis

## Abstract

Cardiac vegetations result from bacterium–platelet adherence, activation and aggregation, and are associated with increased morbidity and mortality in infective endocarditis. The GPIIb/IIIa and FcγRIIa platelet receptors play a central role in platelet adhesion, activation and aggregation induced by endocarditis pathogens such as *Staphylococcus aureus*, but the influence of known polymorphisms of these receptors on the pathogenesis of infective endocarditis is unknown. We determined the GPIIIa platelet antigen Pl^A1/A2^ and FcγRIIa H131R genotype of healthy volunteers (*n* = 160) and patients with infective endocarditis (*n* = 40), and investigated the influence of these polymorphisms on clinical outcome in infective endocarditis and *S. aureus*–platelet interactions in vitro. Platelet receptor genotype did not correlate with development of infective endocarditis, vegetation characteristics on echocardiogram or the composite clinical end-point of embolism, heart failure, need for surgery or mortality (*P* > 0.05 for all), even though patients with the GPIIIa Pl^A1/A1^ genotype had increased in vivo platelet activation (*P* = 0.001). Furthermore, neither GPIIIa Pl^A1/A2^ nor FcγRIIa H131R genotype influenced *S. aureus*-induced platelet adhesion, activation or aggregation in vitro (*P* > 0.05). Taken together, our data suggest that the GPIIIa and FcγRIIa platelet receptor polymorphisms do not influence *S. aureus*–platelet interactions in vitro or the clinical course of infective endocarditis.

## 1 Introduction

Infective endocarditis is a life-threatening condition resulting from infection of native and prosthetic heart valves, implanted medical devices and large vessels [1]. In recent years, *Staphylococcus aureus* has overtaken viridans streptococci as the commonest causative agent, accounting for up to 49% of cases [2,3]. This is worrisome as *S. aureus* infective endocarditis has mortality rates as high as 56% [4], and underlying cardiac risk factors cannot be identified in over 50% of patients [2].

Vegetations containing bacteria, fibrin and activated platelets [5–7], are identified in up to 95% of patients with infective endocarditis, with increased vegetation size associated with embolism and mortality [8–10]. When encased in vegetations, bacteria are able to evade the host immune system and antibiotic therapies, while still being able to grow and disseminate [5–7]. Bacteria stimulate platelet aggregation leading to vegetation formation through the initial binding of microbial surface components recognizing adhesive matrix molecules (MSCRAMMs) to platelet glycoprotein receptors GPIIb/IIIa or GPIb via plasma protein cross-bridges [11]. The subsequent cross-linking of specific immunoglobulins bound to these MSCRAMMs with the FcγRIIa receptor on the platelet surface results in platelet activation, followed by platelet aggregation mediated by the formation of fibrinogen bridges between GPIIb/IIIa receptors [11].

GPIIb/IIIa is a heterodimeric integrin protein linked noncovalently by disulphide bridges [12]. It is the most abundant platelet receptor with 80,000 copies per platelet, and is implicated in platelet aggregation induced by a range of agonists [12]. First described in 1989, the GPIIIa platelet antigen (Pl^A1/A2^) polymorphism consists of a thymine to cytosine substitution at nucleotide 1565 that modulates myosin light chain dephosphorylation, resulting in increased ‘outside-in’ signaling [13,14]. Previous studies have identified enhanced platelet aggregation in response to pharmacological agonists, and increased susceptibility to vascular and immune disease in Pl^A2^ carriers [15–17]. It is feasible that the Pl^A2^ allele is also associated with enhanced bacterium–platelet interactions, vegetation formation and adverse outcome in infective endocarditis.

FcγRIIa is a low affinity IgG-Fc receptor, numbering between 1300 and 8500 on the platelet surface [18,19]. A functional polymorphism resulting from a G to A point mutation leads to an arginine (R) to histidine (H) change at position 131, with codominant expression of both alleles [19,20]. The H131 allotype exhibits increased binding to human IgG_2_ and IgG_3_ and has been associated with the development of systemic lupus erythematosus on meta-analysis [19,20]. However, the influence of this polymorphism on susceptibility to infective endocarditis is unknown.

In as much as bacterium–platelet interactions are central to the pathogenesis of infective endocarditis, we examined the influence of the GPIIIa Pl^A1/A2^ and the FcγRIIa H131R platelet receptor polymorphisms on *S. aureus*-induced platelet adhesion, activation and aggregation in vitro and clinical outcome in patients with infective endocarditis.

## 2 Materials and methods

### 2.1 Subjects

One hundred and sixty healthy volunteers with no evidence of clinically significant co-existing conditions, recent infection or drug therapy were recruited in Edinburgh between July 2007 and March 2009. Forty patients admitted to Edinburgh Royal Infirmary with a diagnosis of infective endocarditis fulfilling the modified Duke criteria [21], were recruited between November 2006 and March 2009. Exclusion criteria included age less than 18 y and inability to provide consent. Written informed consent was obtained from all participants and all studies were conducted following approval from the Lothian NHS Research Ethics Committee and in accordance with the 1964 Declaration of Helsinki.

### 2.2 Clinical outcomes and definitions

Data for patients with infective endocarditis were collected prospectively for the index presentation. The diagnosis of embolism was based on clinical examination and non-invasive radiological examinations were performed at the lead clinician’s discretion. Cutaneous manifestations were excluded, as their etiology is considered multifactorial [22]. Systematic computed tomography surveillance for embolism was not performed. The composite clinical end-point was defined as the presence of embolism, heart failure, need for surgery or death in patients with infective endocarditis within 3 months of diagnosis.

### 2.3 Echocardiography

Echocardiographic images were acquired from transthoracic and/or transesophageal windows performed during the acute phase of the infective endocarditis process using 2-dimensional transducers capable of second and ultra-harmonic imaging, attached to either Vingmed (GE Healthcare, Bedford, UK) or iE33 (Philips, Surrey, UK) platforms. All data were stored digitally. Transthoracic and transesophageal echocardiographic images were independently reviewed on EchoPAC v 7.0.1 for the PC (GE Healthcare Vingmed Ultrasound, Bedford, UK) by two experienced observers (DKD and GJP) who were blinded to subject data including clinical history and platelet receptor genotype. Vegetations were identified as distinctly abnormal echogenic masses attached to the endothelial surface of valves or endocardium. The largest length and width obtainable in any one frame throughout the cardiac cycle was measured for the largest vegetation if more than one vegetation was present [9].

### 2.4 Blood sampling

Venepuncture was performed using a 19-gauge (1.1 mm) needle or a 7 French (2.3 mm) triple-lumen central venous catheter in situ. Care was taken to ensure a smooth blood draw with minimal trauma. Where blood was sampled from a central venous catheter, the initial 10 ml was discarded to minimize sample contamination with heparin. Venous blood (9 ml) was drawn into tubes containing ethylenediamine tetraacetic acid at a final concentration of 1.6 mg/ml for genotyping of platelet receptor polymorphisms in both healthy volunteers and patients with infective endocarditis. Venous blood (3 ml) was drawn into tubes containing 75 μM d-phenylalanyl-l-propyl-l-arginine chloromethylketone (Haematologic Technologies Inc, VT, USA) for determination of platelet activation in patients with infective endocarditis.

Blood samples for isolation of platelet-rich plasma (PRP) for platelet aggregometry and flow cytometry were taken from selected healthy volunteers into 50 ml syringes containing 0.38% (w/v) sodium citrate. For the isolation of washed platelets, blood from healthy subjects was drawn into 50 ml syringes containing 15% (w/v) acid-citrate dextrose (7.32 g/l citric acid anhydrous, 22 g/l sodium citrate, 24.52 g/l dextrose). PRP and washed platelets were generated as described below.

### 2.5 Platelet receptor polymorphism genotyping

Genomic DNA was extracted from blood using the NucliSens easyMag kit (BioMerieux, Basingstoke, UK) according to the manufacturer’s instructions and adjusted to a final concentration of 10 ng/μl in deionized water. The GPIIIa Pl^A1/A2^ (rs5918) and FcγRIIa H131R (rs1801274) polymorphisms were determined with the ABI 7900HT Sequence Detection System (Applied Biosystems, Warrington, UK) with proprietary oligonucleotide sequences and TaqMan^®^ probes (Applied Biosystems, Warrington, UK).

### 2.6 Generation of PRP and washed platelets

Citrated blood from healthy volunteers was centrifuged at 150*g* for 10 min to generate PRP. The PRP fraction was removed and an aliquot was further centrifuged at 1500*g* for 10 min to generate platelet-poor plasma (PPP). Autologous PPP was used to standardize PRP to a platelet count of 200 × 10^9^/l after the platelet count was determined on a Coulter^®^ A.T™ series analyzer (Beckman Coulter Inc., High Wycombe, UK).

For preparation of washed platelets, PRP was generated from blood anticoagulated with acid-citrate dextrose and adjusted to a pH of 6.5 with acid-citrate dextrose, prior to addition of 1 μM prostaglandin E_1_ (Sigma, Dorset, UK) and 1 U/ml apyrase (Sigma, Dorset, UK). Samples were centrifuged at 720*g* for 10 min to obtain a platelet pellet, which was gently resuspended in modified HEPES-Tyrode’s (JNL) buffer (6 mM dextrose, 130 mM NaCl, 9 mM NaHCO_3_, 10 mM sodium citrate, 10 mM TRIS-base, 3 mM KCl, 0.8 mM KH_2_PO_4_, 0.9 mM MgCl_2_; pH 7.4) [23]. Washed platelet suspensions were standardized to a platelet count of 200 × 10^9^/l in JNL buffer, with addition of 1 mg/ml human fibrinogen (Calbiochem, Nottingham, UK) and 2 mM Ca^2+^.

All platelet preparations were used within 2.5 h of venepuncture.

### 2.7 Bacterial strain preparation

*S. aureus* strain Newman and clinical infective endocarditis *S. aureus* strains 207 and 209 [25] were grown to stationary and exponential phase in brain–heart infusion (BHI) broth (Oxoid, Basingstoke, UK) at 37 °C with constant rotation at 200 rpm. Bacterial growth was assessed by measurement of the optical density at 600 nm (OD_600_) using a spectrophotometer (Cecil Aurius CE2021, Thistle Scientific Ltd., Glasgow, UK). Stationary phase was defined as an OD_600_ value greater than 12 following culture for 16 h, while exponential phase was defined as an OD_600_ value of between 0.5 and 0.9.

For platelet activation and aggregation studies, bacterial cultures were washed twice in phosphate-buffered saline (PBS) and resuspended to an OD_600_ value of 1.6. For *S. aureus*–platelet adhesion studies, cultures were washed twice in cold 0.05 M Tris–HCl buffer containing 0.1 M NaCl and 0.02 M ethylenediamine tetraacetic acid, pH 7.25. Bacteria were labeled with 100 μg/ml of Hoechst 33342 (Sigma, Dorset, UK) before incubation in the dark for 2 h at 4 °C, followed by twice washing in Tyrode’s solution (137 mM NaCl, 2.7 mM KCl, 1 mM MgCl_2_, 1.8 mM CaCl_2_, 0.2 mM Na_2_HPO_4_, 12 mM NaHCO_3_, 5.5 mM dextrose, pH 7.4) and resuspension to an OD_600_ of 1.6 [24].

### 2.8 Platelet aggregometry

Platelet aggregometry was performed as described previously [25]. Briefly, 5 × 10^7^ bacterial cells (in 25 μl PBS) were added to 4.5 × 10^7^ platelets (in 225 μl PRP or washed platelets) in siliconized glass cuvettes with constant stirring at 37 °C. Platelet aggregation was assayed by light transmission in a PAP-4 aggregometer (Bio-Data, Alpha Laboratories, Eastleigh, UK) in triplicate. PPP and JNL buffer were used as the 100% light transmission references for PRP and washed platelets respectively. The pharmacological protease activated receptor type 1 agonist SFLLRN–NH_2_ (0.1 μM; Clinalfa, Läufelfingen, Switzerland) and adenosine diphosphate (ADP, 5 μM; Trinity Biotech, Wicklow, Ireland) were used as agonist positive controls. The lag time to platelet aggregation was measured as the time from addition of agonist to the initiation of platelet aggregation up to a 25 min limit. The maximal percentage platelet aggregation was also determined.

### 2.9 Flow cytometry

#### 2.9.1 Bacterium–platelet adhesion

*S. aureus*–platelet adhesion was measured following incubation of 9 × 10^7^ Hoechst 33342-labeled bacterial cells with 9 × 10^6^ platelets in PRP at room temperature for 5 min, followed by labeling with 1% fluorescein isothiocyanate (FITC)-conjugated mouse anti-human CD42a antibody (Serotec, Kidlington, UK) for 30 min, and fixing with 1 ml 1% (v/v) paraformaldehyde. Fluorescent data were determined within 24 h of fixation using a FACSVantage SE cell sorter (Becton Dickinson, Oxford, UK) with FACSDiVa software (Becton Dickinson, Oxford, UK). One hundred thousand platelets were collected for each sample and distilled water, flow buffer (Becton Dickinson, Oxford, UK) and Tris buffer served as negative controls. *S. aureus* bound to platelets were identified as cells positive for both Hoechst and FITC fluorescent signals, and data were analyzed using FlowJo v 8.8.6 (Tree Star Inc., OR, USA) to determine the percentage of platelets bound to *S. aureus* and the percentage of *S. aureus* cells bound to platelets [24].

#### 2.9.2 In vivo and in vitro platelet activation

Within 5 min of transfer into a d-phenylalanyl-l-propyl-l-arginine chloromethylketone tube, blood was labeled at room temperature with the appropriate monoclonal antibodies for determination of in vivo platelet activation [26,27]. Briefly, mouse anti-human CD14-phycoerythrin (PE; Inverness Medical, Stockport, UK) and anti-CD42a-FITC were added to 60 μl of blood at a 1:40 dilution for detection of platelet–monocyte aggregates (PMA). Anti-CD42a-FITC and mouse anti-human CD62P-PE (Serotec, Kidlington, UK) at a 1:2 dilution were used to label 5 μl of blood for detection of platelet P-selectin expression. The appropriate isotope mouse IgG_1_ antibodies (Serotec, Kidlington, UK) and flow buffer served as negative controls. Following 20 min of immunolabelling, PMA samples were fixed with 500 μl FACS lyse solution (Becton Dickinson, Oxford, UK) and P-selectin samples with 1.425 ml 1% (v/v) paraformaldehyde.

Platelet activation was quantified within 24 h of sample fixation using dual-channel flow cytometry (FACSCalibur, Becton Dickinson, Oxford, UK) with at least 2000 monocytes and 7500 platelets collected for each assay. PMAs were defined as monocytes expressing CD14 that were also positive for CD42a, and P-selectin-expressing platelets were defined as platelets that were positive for CD62P. Data were acquired and analyzed using CellQuest software (Becton Dickinson, Oxford, UK), and results expressed as a percentage of the monocyte or platelet cell population respectively.

For determination of agonist-induced platelet P-selectin expression in vitro, bacterial suspensions were added to PRP at a ratio of 10:1, incubated for 30 min at room temperature, and labeled with 2% anti-CD42a-FITC, anti-CD62P-PE or the appropriate IgG_1_ isotype control for 30 min. Samples were fixed with 1 ml 1% (v/v) paraformaldehyde, and platelet P-selectin expression was determined as described above using a FACScan flow cytometer (Becton Dickinson, Oxford, UK). SFLLRN–NH_2_ (1 μM) was used as a positive control.

### 2.10 Immobilized plasma protein adherence assays

Adherence of bacteria to immobilized fibrinogen and fibronectin was performed as previously described [25,28]. Briefly, 96-well flat-bottomed plates (Nunc, Loughborough, UK) were coated with serial dilutions of 100 μl of 0–50 μg/ml human fibrinogen or bovine fibronectin in PBS, and incubated at 4 °C overnight. Plates were thrice washed with 100 μl PBS, and then incubated with 100 μl of 2 mg/ml filter-sterilised bovine serum albumin (Sigma, Dorset, UK) for 1 h at 37 °C. *S. aureus* cells (1.25 × 10^8^) were added to each well and incubated for 2 h at 37 °C under constant rotation, fixed with 100 μl 25% (v/v) formaldehyde (VWR, Lutterworth, UK) for 30 min at room temperature, stained with 100 μl 0.5% (w/v) crystal violet (Sigma, Dorset, UK) for 1 min, and washed three times with 100 μl PBS between each fixing or staining stage. The absorbance at 590 nm (A_590_) was determined in an enzyme-linked immunosorbent assay (ELISA) plate reader (VERSAmax microplate reader, Molecular Devices, Wokingham).

### 2.11 Statistical analysis

Data analysis was performed using SPSS v. 16.0 (SPSS Inc, Chicago, IL, USA) for Macintosh. The chi-squared test was used to compare polymorphism frequencies and the normality of data distribution was determined using the Shapiro–Wilk test. The Kruskal–Wallis and Mann–Whitney *U* tests were used to analyze the relationship between platelet receptor genotype and numerical data, while Fisher’s exact and the chi-squared tests were used to determine the correlation between platelet receptor polymorphisms and categorical variables. Statistical significance was taken as two-sided *P* < 0.05 for all analyses. Values are expressed as mean ± standard deviation.

## 3 Results

### 3.1 Characteristics of the study population

Clinical characteristics of 40 recruited patients with infective endocarditis (75% male, 56 ± 17 y) are outlined in Table 1. Patients were recruited into the study 12 ± 15 d after diagnosis. The mitral or aortic valves were affected in 87% of cases, with streptococci and staphylococci collectively accounting for 70% of cases of infective endocarditis. Just over a third of patients had underlying valvular heart disease or prosthetic valves, 15% had a past history of infective endocarditis and 10% were intravenous drug abusers. Vegetations were identified in 78% of subjects, and vegetation dimensions were measured in 97% of patients using transthoracic or transesophageal echocardiographic images acquired 7 ± 8 d after diagnosis. Clinically detected emboli were present in 38% of cases, heart failure in 30%, 63% required surgical treatment and the mortality rate in our patient cohort was 23%. The composite clinical end-point of embolism, heart failure, need for surgery and heart failure was present in 75% of patients. Out of the 160 healthy volunteers, 33% were male with a mean age of 30 ± 10 y.

### 3.2 Susceptibility to infective endocarditis

We determined the distribution of the GPIIIa Pl^A1/A2^ and FcγRIIa H131R polymorphisms in patients with infective endocarditis and all allelic variants were in Hardy–Weinberg equilibrium (*P* = 0.686 and *P* = 0.770, respectively). The distribution of GPIIIa Pl^A1/A2^ and FcγRIIa H131R platelet receptor genotype did not vary between patients with infective endocarditis and healthy volunteers (*P* = 0.675 and *P* = 0.774 respectively; Table 2), and were consistent with findings from previous studies of platelet receptor polymorphisms [13,19,28].

### 3.3 In vivo platelet activation and clinical outcome

We analyzed the association of GPIIIa Pl^A1/A2^ and FcγRIIa H131R genotype with in vivo platelet activation and clinical characteristics in patients with infective endocarditis. Given the modest study population, a composite clinical end-point was used.

**Fig. 1:**
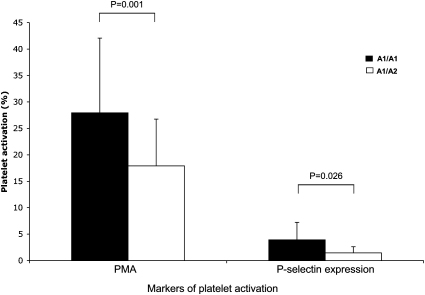
Association between GPIIIa Pl^A1/A2^ genotype and platelet activation in patients with infective endocarditis. Data for the one Pl^A2/A2^ patient are not depicted. PMA, platelet–monocyte aggregates. *P* values were determined using the Mann–Whitney *U* test.

Baseline demographic variables for subjects stratified by GPIIIa Pl^A1/A2^ genotype are outlined in Table 3. As only one subject had the Pl^A2/A2^ genotype, data for this individual were excluded from all analyses. Apart from an association of the GPIIIa Pl^A1/A1^ genotype with infection by staphylococci or streptococci (*P* = 0.030; Table 3), the GPIIIa Pl^A1/A2^ polymorphism did not influence background factors in patients with infective endocarditis. The GPIIIa Pl^A1/A1^ genotype was associated with increased in vivo platelet activation (*P* = 0.001 for PMA and *P* = 0.026 for platelet P-selectin expression; Fig. 1) and reduced embolism (*P* = 0.027; Table 3). However, there were no correlations of the GPIIIa Pl^A1/A2^ polymorphism with vegetation characteristics or the composite clinical end-point of embolism, heart failure, need for surgery or mortality (*P* > 0.05 for all).

Demographic variables for patients with infective endocarditis, categorized by FcγRIIa H131R genotype, are depicted in Table 4. Apart from an association of the R131 allele with solitary aortic valve infection (*P* = 0.005), clinical characteristics did not vary with FcγRIIa H131R genotype. Platelet activation, measured as both PMA and platelet P-selectin expression, did not vary with FcγRIIa H131R genotype (*P* = 0.656 and *P* = 0.891 respectively; data not shown). Although the H/R genotype was associated with reduced need for surgery (*P* = 0.005; Table 4) and the H131 allele with the development of heart failure (*P* = 0.024; Table 4), there were no correlations between FcγRIIa H131R genotype, vegetation characteristics or the composite clinical end-point.

### 3.4 Bacterium–platelet interactions in vitro

**Fig. 2:**
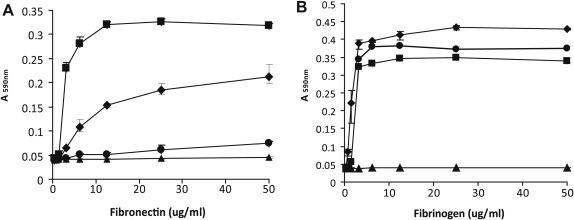
Adherence of *S. aureus* strains to immobilized plasma proteins. Strains Newman (♦), 207 (●), or 209 (▪), were grown to exponential phase and incubated with doubling dilutions of immobilized bovine fibronectin (A), or grown to stationary phase and incubated with doubling dilutions of immobilized human fibrinogen (B). Adherence of strain Newman to wells incubated with BSA only was used to determine background levels (▴).

In order to investigate the influence of platelet receptor polymorphisms on *S. aureus*–platelet interactions, we examined *S. aureus*–platelet adhesion, activation and aggregation in vitro using platelets from healthy volunteers of different GPIIIa Pl^A1/A2^ and FcγRIIa H131R genotype (Table 2). In total, six different bacterial preparations, including *S. aureus* strain Newman and two clinical isolates from infective endocarditis (strains 207 and 209), grown to both exponential and stationary phases of growth, were used in platelet interaction studies. *S. aureus* strain 207 grown to stationary phase did not induce platelet aggregation within 25 min and was therefore not utilized as an agonist in further *S. aureus*–platelet interaction studies. The *S. aureus* strains employed exhibited differences in the capacity to adhere to immobilized fibrinogen and fibronectin in vitro (Fig. 2). In the exponential phase of growth, strain Newman demonstrated weak binding to fibronectin, and strain 207 did not adhere to fibronectin at all. In stationary phase, all three strains adhered to fibrinogen with the highest affinity exhibited by strain Newman (Fig. 2).

Platelet aggregometry was performed with PRP obtained from 88 of the 160 healthy volunteers (40% male, 30 ± 9 y), selected to represent different GPIIIa Pl^A1/A2^ and FcγRIIa H131R genotypes (Table 2). Results obtained using Pl^A2/A2^ platelets were excluded from all analyses given the small number of subjects in this group (*n* = 4) and the highly variable data (Table 5). An association of GPIIIa Pl^A1/A1^ genotype with reduced lag time to aggregation (*P* = 0.038) was apparent for one of the six bacterial agonists examined (exponential phase Newman; Table 5), while an increase in percentage aggregation of GPIIIa Pl^A1/A1^ platelets (*P* = 0.033) was observed for another bacterial agonist (stationary phase 209; Table 5). Exponential phase 209 was associated with reduced percentage aggregation of platelets with the FcγRIIa R/R genotype (*P* = 0.043; Table 6). For all other bacterial agonist preparations, there were no differences in aggregation of platelets of different GPIIIa Pl^A1/A2^ or FcγRIIa H131R genotype in PRP (Tables 5 and 6), suggesting that platelet receptor polymorphisms do not have a broad influence on *S. aureus*-induced platelet aggregation. Notably, percentage platelet aggregation did not vary with platelet receptor genotype for the pharmacological agonists ADP and SFLLRN–NH_2_ (Tables 5 and 6).

In order to investigate the possibility that differences in plasma protein and IgG concentration could influence sensitivity to platelet aggregation [29,30], platelet aggregometry was repeated with washed platelets isolated from 31 healthy volunteers (52% male, 33 ± 9 y) selected on the basis of their platelet receptor genotype (Table 2). There were no significant correlations between platelet receptor genotype and aggregation of washed platelets induced by *S. aureus* and pharmacological agonists (Tables 5 and 6), implying that the small number of significant associations observed for specific bacterial agonists in PRP may have been due to differences in plasma protein concentrations in human volunteers.

Finally, in order to determine whether the GPIIIa Pl^A1/A2^ and FcγRIIa H131R polymorphisms influence *S. aureus*–platelet adhesion or activation distinct from aggregation, flow cytometry was performed using blood from the same 31 representative individuals used for washed platelet aggregation analysis (Table 2). We discovered that GPIIIa Pl^A1/A2^ and FcγRIIa H131R genotype did not influence *S. aureus*–platelet binding or *S. aureus*-induced platelet activation (Tables 5 and 6).

## 4 Discussion

Previous studies have identified associations between platelet receptor polymorphisms and susceptibility to vascular and immune disease [15,16,19,20]. This is the first study to investigate the association between platelet receptor genotype, *S. aureus*–platelet interactions and clinical outcome in infective endocarditis. Overall, our data suggest that the GPIIIa Pl^A1/A2^ and FcγRIIa H131R platelet receptor polymorphisms do not have a major influence on S*. aureus*–platelet adhesion, activation and aggregation in vitro or prognosis in infective endocarditis.

The GPIIIa Pl^A1/A2^ and FcγRIIa H131R platelet receptor polymorphisms did not influence *S. aureus*–platelet adhesion or activation in vitro. However, highly variable strain-dependent effects on *S. aureus*-induced platelet aggregation were observed, highlighting the dynamic interaction between host and pathogen that may influence the outcome of infection [11]. Considerable differences in the capacity to induce platelet aggregation exist among *S. aureus* clinical isolates, likely reflecting variation in MSCRAMM expression [23,25,31]. For example, strain 209 induced more rapid platelet aggregation in the exponential phase compared to the stationary phase of growth, while the converse was true for strain Newman (Tables 5 and 6). This is consistent with our knowledge of the predominant role of fibronectin-binding proteins (Fnbps), in *S. aureus*-induced platelet aggregation during the exponential phase of growth, as strain 209 adhered strongly to fibronectin whereas strain Newman does not express functional Fnbps and binds fibronectin weakly (Fig. 2) [32]. In addition, strain 207 did not induce rapid platelet aggregation in exponential phase, consistent with its complete lack of fibronectin-binding activity (Fig. 2). In the stationary phase of growth, the fibrinogen-binding protein clumping factor A (ClfA) is regarded as the predominant surface protein involved in platelet aggregation [33]. However, strains 207 and 209 had long lag times to platelet aggregation in stationary phase, in spite of their capacity to bind to fibrinogen (Fig. 2), but both strains demonstrated lower adherence compared to strain Newman, possibly reflecting lower levels of expression of ClfA on the bacterial cell surface.

The associations between GPIIIa Pl^A1/A2^ and FcγRIIa H131R genotype and *S. aureus*-induced platelet aggregation in PRP were lost when washed platelets were used, suggesting that variation in the concentration of immunoglobulins or plasma proteins such as fibrinogen, fibronectin or von Willebrand factor may have influenced our results [11,29].

Our data are consistent with previous studies that have demonstrated a lack of effect of the FcγRIIa H131R genotype on shear-induced platelet activation and platelet aggregation induced by both pharmacological agonists and streptococci [18,30]. However, our observations contrast with those of large studies that have demonstrated increased platelet activation and aggregation in response to ADP and epinephrine in Pl^A2^ carriers. This may be due to the use of bacterial agonists and saturating concentrations of pharmacological agonists in the current study, and the paucity of subjects carrying the GPIIIa Pl^A2/A2^ genotype [17].

Consistent with our in vitro experiments and large studies that have examined the role of platelet receptor polymorphisms in thrombosis, immune disease and intravascular device infection, GPIIIa Pl^A1/A2^ and FcγRIIa H131R genotype did not influence the development of infective endocarditis, vegetation characteristics or the composite clinical end-point of embolism, heart failure, need for surgery or mortality [16,34–36]. Notably, the demographics of our infective endocarditis study population were similar to those observed in large multi-center published studies [2,37], but rates of staphylococcal endocarditis and vegetation formation were lower than expected, possibly due to a reduced prevalence of *S. aureus* infection in comparison to the USA [2,4,37]. Rates of heart failure, embolism and mortality were comparable to other published research, while rates of surgery were higher than reported by Murdoch et al., most likely attributable to the Edinburgh Royal Infirmary being a tertiary referral center for cardiothoracic surgery [2,38].

In contrast to our in vitro analysis, we identified increased platelet activation in patients with infective endocarditis homozygous for the Pl^A1^ allele, suggesting that the Pl^A1/A1^ genotype may influence bacterium–platelet interactions in vivo. However, an effect of the differing demographics of our study populations, which included predominantly older male infective endocarditis patients and a cohort of mostly younger female healthy volunteers, cannot be ruled out, particularly as it is known that platelet activation increases with age and in female subjects [39,40]. It is also possible that the low shear environment encountered during platelet aggregometry in vitro, in contrast to the high shear environment in vivo, may influence *S. aureus*–platelet interactions [41]. Furthermore, it is possible that the growth of *S. aureus* in human blood in vivo, as opposed to nutrient-rich culture medium in vitro, may influence the expression of MSCRAMMs implicated in bacterium-induced platelet activation [42,43].

Finally, it is important to note that the current study had a modest patient population size, particularly with regards to the Pl^A2/A2^ genotype. A larger study would also enable correction for possible confounding factors such as age, gender, ethnicity, antibiotic treatment, platelet receptor density, source of infection and underlying cardiac and bacteremic risk factors for infective endocarditis [22]. In particular, correction for pathogen-related factors such as route of acquisition and bacterial species would be desirable. Nevertheless, the combination of approaches employed here, including the analysis of samples from well-defined clinical cases of infective endocarditis and in vitro platelet functional analyses, strongly suggest that the GPIIIa Pl^A1/A2^ and FcγRIIa H131R platelet receptor polymorphisms do not influence *S. aureus*–platelet interactions in vitro and do not constitute risk factors for infective endocarditis.

## Conflict of interest statement

SD is currently employed by GlaxoSmithKline, UK.

## Figures and Tables

**Table 1:** Clinical characteristics of 40 recruited patients with infective endocarditis.

Clinical feature	Number (%)
Age, y	56 ± 17
Male	30 (75)
Time from diagnosis to study participation	12 ± 15 d
Type of valve	
Native	26 (65)
Prosthetic	14 (35)
Bioprosthetic	8
Mechanical	6
Valvular heart disease	14 (35)
Congenital heart disease	8
Bicuspid aortic valve	4
Rheumatic heart disease	1
Degenerative valve	1
Previous infective endocarditis	6 (15)
Intravenous drug abuse	4 (10)
Affected valve(s)	
Mitral	16 (40)
Aortic	13 (32)
Mitral + aortic	6 (15)
Tricuspid ± mitral or aortic	4 (10)
Pulmonary	1 (3)
Organism(s)	
Streptococci	15 (37)
Viridans streptococci	11
Non-viridans streptococci	4
Staphylococci	11 (28)
*Staphylococcus aureus*	8
Coagulase-negative staphylococci	3
Streptococci + staphylococci	2 (5)
Enterococci	2 (5)
Other^a^	7 (17)
Culture-negative	3 (8)
Vegetation	
Present	31 (78)
Length, mm^b^	15 ± 8
Width, mm^b^	9 ± 4
Surgery	25 (63)
Replacement	19
Repair	5
Replacement + repair	1
Embolic phenomena^c^	15 (38)
Cerebral	8
Splenic	3
Pulmonary	2
Spinal	2
Renal	2
Heart failure	12 (30)
Death	9 (23)
Composite clinical end-point^d^	30 (75)

a Other organisms isolated include *Aerococcus urinae*, *Aerococcus viridans*, *Gemella sanguinis*, *Granulicatella adiacens*, *Haemophilus parainfluenzae*, *Propionibacterium acnes* and *Serratia marcescens* (*n* = 1 for each).

b Vegetation characteristics were determined for 30 subjects.

c 2 subjects had more than one site of embolism.

d The clinical end-point represents a composite of embolism, heart failure, need for surgery and mortality.

**Table 2:** Frequencies of the platelet receptor GPIIIa Pl^A1/A2^ and FcγRIIa H131R polymorphisms in 40 patients with infective endocarditis compared to 160 healthy volunteers.

Platelet receptor genotype	Infective endocarditis patients, *n* = 40 (%)	Healthy volunteers, *n* = 160 (%)	*P* value^a^	Healthy volunteers *n* = 88^b^	Healthy volunteers *n* = 31^c^
GPIIIa Pl^A1/A2^					
Pl^A1/A1^	29 (72%)	126 (78%)	0.675	58	17
Pl^A1/A2^	10 (25%)	30 (19%)	26	10
Pl^A2/A2^	1 (3%)	4 (3%)	4	4

FcγRIIa H131R					
H/H	11 (28%)	39 (24%)	0.774	23	7
H/R	19 (47%)	72 (45%)	40	16
R/R	10 (25%)	49 (31%)	25	8

a The *P* value was determined using the chi-squared test.

b Platelet receptor genotype for healthy volunteers selected for platelet aggregation studies using PRP.

c Platelet receptor genotype for healthy volunteers selected for flow cytometric studies and platelet aggregation studies using washed platelets.

**Table 3:** Baseline demographics and clinical outcome in 39 patients with infective endocarditis stratified by the GPIIIa Pl^A1/A2^ platelet receptor genotype.

	GPIIIa Pl^A1/A2^ genotype		*P* value^a^
A1/A1, *n* = 29	A1/A2, *n* = 10
Age (y)	56 ± 17	56 ± 17	0.949
Male (%)	22 (76)	8 (80)	1.000
Native valve (%)	20 (69)	6 (60)	0.704
Valvular heart disease (%)	8 (28)	5 (50)	0.254
Previous infective endocarditis (%)	2 (7)	3 (30)	0.096
Intravenous drug abuser (%)	3 (10)	1 (10)	1.000
Affected valve (%)			
Mitral	11 (38)	4 (40)	0.524
Aortic	10 (35)	3 (30)
Mitral + aortic	5 (17)	1 (10)
Tricuspid ± mitral/aortic	3 (10)	1 (10)
Pulmonary	0	1 (10)
Presence of vegetations (%)	22 (76)	8 (80)	1.000
Vegetation length, mm^b^	14 ± 5	20 ± 12	0.166
Vegetation width, mm^b^	9 ± 4	9 ± 3	0.946
Organism (%)			
Streptococci	13 (45)	2 (20)	0.030
Staphylococci	10 (35)	1 (10)
Streptococci + staphylococci	2 (7)	0 (0)
Enterococci	1 (3)	1 (10)
Other	2 (7)	5 (50)
Culture negative	1 (3)	1 (10)
Surgery (%)	18 (62)	7 (70)	0.721
Embolic phenomena (%)	8 (28)	7 (70)	0.027
Heart failure (%)	10 (34)	1 (10)	0.228
Death (%)	6 (21)	3 (30)	0.669
Composite clinical end-point (%)^c^	22 (73%)	8 (80%)	1.000

a *P* values were determined using the Mann–Whitney *U* test for numerical variables, and Fisher’s exact test or the chi-squared test for categorical variables. Data for the one Pl^A2/A2^ subject are not depicted and were excluded from all analyses.

b Data on vegetation characteristics were available for 29 of the 30 subjects with vegetations.

c The clinical end-point represents a composite of embolism, heart failure, need for surgery and mortality.

**Table 4:** Baseline demographics and clinical outcome in 40 patients with infective endocarditis stratified by the FcγRIIa H131R platelet receptor polymorphism.

	FcγRIIa H131R genotype			*P* value^a^
H/H, *n* = 11	H/R, *n* = 19	R/R, *n* = 10
Age (y)	56 ± 13	59 ± 18	49 ± 21	0.470
Male (%)	6 (55)	15 (79)	9 (90)	0.149
Native valve (%)	8 (73)	10 (53)	8 (80)	0.279
Valvular heart disease (%)	7 (64)	5 (26)	2 (20)	0.061
Previous infective endocarditis (%)	3 (27)	2 (11)	1 (10)	0.408
Intravenous drug abuser (%)	0	3 (16)	1 (10)	0.381
Affected valve (%)				
Mitral	5 (45)	7 (37)	4 (40)	0.005
Aortic	0	11 (58)	2 (20)
Mitral + aortic	5 (45)	0	1 (10)
Tricuspid ± mitral/aortic	1 (10)	1 (5)	2 (20)
Pulmonary	0	0	1 (10)
Presence of vegetations (%)	9 (82)	14 (74)	8 (80)	0.856
Vegetation length, mm^b^	21 ± 14	13 ± 5	16 ± 7	0.166
Vegetation width, mm^b^	8 ± 2	8 ± 4	10 ± 5	0.946
Organism (%)				
Streptococci	4 (37)	6 (32)	5 (50)	0.545
Staphylococci	2 (18)	6 (32)	3 (30)
Streptococci + staphylococci	1 (9)	1 (5)	0
Enterococci	2 (18)	0	0
Other	1 (9)	4 (21)	2 (20)
Culture negative	1 (9)	2 (10)	0
Surgery (%)	10 (91)	7 (37)	8 (80)	0.005
Embolic phenomena (%)	2 (18)	8 (42)	5 (50)	0.274
Heart failure (%)	6 (55)	6 (32)	0	0.024
Death (%)	4 (36)	5 (26)	0	0.118
Composite clinical end-point (%)^c^	10 (91%)	12 (63%)	8 (80%)	0.219

a *P* values were determined using the Kruskal–Wallis test with Mann–Whitney *U* correction for numerical variables and the chi-squared test for categorical variables.

b Data on vegetation characteristics were available for 30 of 31 subjects with vegetations.

c The clinical end-point represents a composite of embolism, heart failure, need for surgery and mortality.

**Table 5:** Correlation between GPIIIa Pl^A1/A2^ genotype and *S. aureus*-induced platelet aggregation, activation and *S. aureus*–platelet binding using blood from selected healthy volunteers.

Agonist	GPIIIa Pl^A1/A2^ genotype			*P* value^a^
A1/A1	A1/A2	A2/A2
**Platelet-rich plasma (*n* = 88)**				
*Lag time, min*				
Newman, stationary	1.92 ± 0.65	1.83 ± 0.47	2.63 ± 1.25	0.965
209, stationary	8.60 ± 3.51	12.44 ± 7.28	6.08 ± 1.41	0.862
Newman, exponential	6.07 ± 1.41	6.60 ± 1.26	6.92 ± 1.03	0.038
207, exponential	9.19 ± 3.36	9.11 ± 3.62	8.31 ± 5.14	0.642
209, exponential	1.94 ± 0.64	1.84 ± 0.50	3.13 ± 1.82	0.768
*Platelet aggregation, %*				
Newman, stationary	67 ± 10	69 ± 7	66 ± 2	0.809
209, stationary	68 ± 8	64 ± 10	63 ± 33	0.033
Newman, exponential	69 ± 12	60 ± 19	76 ± 7	0.091
207, exponential	66 ± 15	66 ± 13	70 ± 16	0.595
209, exponential	63 ± 9	63 ± 14	67 ± 4	0.642
ADP	69 ± 12	74 ± 12	70 ± 6	0.074
SFLLRN–NH_2_	68 ± 12	69 ± 13	69 ± 7	0.762
**Washed platelets (*n* = 31)**				
*Lag time, min*				
Newman, stationary	3.00 ± 0.90	2.89 ± 1.26	9.21 ± 10.56	0.553
209, stationary	10.20 ± 8.73	9.10 ± 9.18	13.66 ± 7.88	0.418
Newman, exponential	8.65 ± 8.02	9.05 ± 9.16	21.01 ± 7.99^b^	0.829
207, exponential	25^c^	19.24 ± 8.78	22.93 ± 4.14^b^	N/A^d^
209, exponential	7.74 ± 5.48	8.74 ± 7.43	16.32 ± 10.10	0.979
*Platelet aggregation, %*				
Newman, stationary	82 ± 4	80 ± 6	78 ± 5	0.590
209, stationary	73 ± 12	77 ± 7	73 ± 17	0.692
Newman, exponential	66 ± 16	75 ± 6	78^b^	0.314
209, exponential	71 ± 12	72 ± 8	75 ± 1	0.830
ADP	29 ± 20	36 ± 17	12 ± 16	0.215
SFLLRN–NH_2_	71 ± 19	75 ± 5	64 ± 22	0.306
**Agonist-induced platelet activation, % (*n* = 31)**				
Newman, stationary	55 ± 29	49 ± 25	43 ± 33	0.711
209, stationary	26 ± 11	18 ± 11	24 ± 6	0.093
Newman, exponential	36 ± 20	40 ± 19	31 ± 16	0.604
207, exponential	24 ± 15	18 ± 12	25 ± 11	0.443
209, exponential	28 ± 17	20 ± 12	21 ± 4	0.223
SFLLRN–NH_2_	82 ± 14	83 ± 6	78 ± 14	0.660
**Percentage of platelets bound to *S. aureus*, % (*n* = 31)**				
Newman, stationary	84 ± 21	85 ± 26	91 ± 8	0.792
209, stationary	86 ± 22	86 ± 23	92 ± 8	0.874
Newman, exponential	58 ± 27	53 ± 26	70 ± 31	0.751
207, exponential	78 ± 25	74 ± 28	77 ± 20	0.426
209, exponential	57 ± 25	52 ± 28	58 ± 19	0.598
**Percentage of *S. aureus* cells bound to platelets, % (*n* = 31)**				
Newman, stationary	49 ± 10	44 ± 15	56 ± 9	0.312
209, stationary	44 ± 12	38 ± 12	48 ± 12	0.200
Newman, exponential	22 ± 12	18 ± 11	25 ± 8	0.711
207, exponential	24 ± 10	21 ± 8	23 ± 3	0.339
209, exponential	18 ± 11	14 ± 7	17 ± 3	0.419

Percentage platelet aggregation in response to strain 207 grown to exponential phase could not be analyzed when using washed platelets, as aggregation was only observed with platelets isolated from 2 subjects. Data for *S. aureus* strain 207 at stationary phase of growth were not analyzed as platelet aggregation was not observed in response to this agonist.

a *P* values were determined using the Mann–Whitney *U* test comparing the Pl^A1/A1^ to the Pl^A1/A2^ genotype.

b Only one Pl^A2/A2^ subject’s washed platelets aggregated in response to *S. aureus* strains Newman and 207 at the exponential phase of growth.

c Lag times of 25 min represent absence of platelet aggregation.

d Statistical analyses could not be performed as platelet aggregation was not observed with Pl^A1/A1^ platelets in response to strain 207 at the exponential phase of growth.

**Table 6:** Correlation between FcγRIIa H131R genotype and *S. aureus*-induced platelet aggregation, activation and *S. aureus*–platelet binding using blood from selected healthy volunteers.

Agonist	FcγRIIa H131R genotype			*P* value^a^
H/H	H/R	R/R
**Platelet-rich plasma (*n* = 88)**				
*Lag time, min*				
Newman, stationary	1.82 ± 0.58	1.94 ± 0.68	2.02 ± 0.69	0.384
209, stationary	8.02 ± 3.69	8.31 ± 3.85	9.91 ± 4.00	0.287
Newman, exponential	5.90 ± 1.61	6.67 ± 1.29	5.92 ± 1.07	0.077
207, exponential	9.28 ± 3.64	9.18 ± 3.60	8.87 ± 3.23	0.765
209, exponential	1.86 ± 0.51	1.92 ± 0.64	2.16 ± 1.01	0.401
*Platelet aggregation, %*				
Newman, stationary	69 ± 9	68 ± 8	65 ± 10	0.351
209, stationary	68 ± 9	67 ± 10	64 ± 14	0.611
Newman, exponential	70 ± 14	65 ± 17	66 ± 9	0.285
207, exponential	69 ± 10	64 ± 17	66 ± 13	0.576
209, exponential	65 ± 6	65 ± 8	57 ± 15	0.043
ADP	73 ± 11	70 ± 13	69 ± 11	0.206
SFLLRN–NH_2_	65 ± 15	70 ± 12	68 ± 9	0.275
**Washed platelets (*n* = 31)**				
*Lag time, min*				
Newman, stationary	3.26 ± 0.66	3.19 ± 1.18	5.42 ± 7.96	0.949
209, stationary	9.00 ± 8.18	9.59 ± 8.13	12.81 ± 10.31	0.948
Newman, exponential	11.98 ± 10.44	8.74 ± 8.19	12.61 ± 10.42	0.328
207, exponential	23.62 ± 3.38	22.85 ± 5.98	22.82 ± 6.18	0.536
209, exponential	9.57 ± 7.90	9.29 ± 7.23	8.68 ± 7.26	0.332
*Platelet aggregation, %*				
Newman, stationary	81 ± 3	79 ± 5	85 ± 4	0.066
209, stationary	75 ± 6	73 ± 14	77 ± 8	0.385
Newman, exponential	69 ± 5	75 ± 5	56 ± 22	0.207
209, exponential	76 ± 3	69 ± 13	74 ± 5	0.251
ADP	33 ± 31	29 ± 16	26 ± 19	0.877
SFLLRN–NH_2_	75 ± 7	71 ± 7	68 ± 18	0.622
**Agonist-induced platelet activation, % (*n* = 31)**				
Newman, stationary	64 ± 29	54 ± 27	40 ± 25	0.294
209, stationary	27 ± 12	22 ± 12	23 ± 9	0.834
Newman, exponential	49 ± 24	38 ± 16	25 ± 15	0.059
207, exponential	31 ± 22	19 ± 11	22 ± 10	0.450
209, exponential	34 ± 23	23 ± 14	21 ± 6	0.410
SFLLRN–NH_2_	78 ± 23	84 ± 6	79 ± 11	0.473
**Percentage of platelets bound to *S. aureus*, % (*n* = 31)**				
Newman, stationary	87 ± 23	87 ± 18	80 ± 26	0.456
209, stationary	84 ± 28	90 ± 16	82 ± 25	0.544
Newman, exponential	56 ± 30	60 ± 29	57 ± 34	0.964
207, exponential	75 ± 31	81 ± 21	70 ± 27	0.476
209, exponential	51 ± 27	59 ± 23	51 ± 30	0.730
**Percentage of *S. aureus* cells bound to platelets, % (*n* = 31)**				
Newman, stationary	52 ± 9	48 ± 13	47 ± 14	0.768
209, stationary	47 ± 13	42 ± 12	40 ± 12	0.550
Newman, exponential	21 ± 6	22 ± 14	19 ± 9	0.896
207, exponential	20 ± 6	25 ± 10	22 ± 7	0.699
209, exponential	17 ± 1	18 ± 11	15 ± 5	0.591

Percentage platelet aggregation in response to strain 207 grown to exponential phase could not be analyzed when using washed platelets, as aggregation was only observed with platelets isolated from 2 subjects. Data for *S. aureus* strain 207 at stationary phase of growth were not analyzed as platelet aggregation was not observed in response to this agonist.

a *P* values were determined using the Kruskal–Wallis test.
